# Impact of Multilayer Coatings on the Mechanical and Durability Performance of FRCM Composites

**DOI:** 10.3390/polym18091130

**Published:** 2026-05-04

**Authors:** Ali Çopuroğlu, Bekir Yilmaz Pekmezci

**Affiliations:** Faculty of Civil Engineering, Istanbul Technical University, 34469 Istanbul, Türkiye; pekmezcib1@itu.edu.tr

**Keywords:** FRCM, TRC, textile coating, multilayer coating, tensile strength, textile-matrix interface, durability, alkaline exposure, fabric reinforced cementitious matrices, textile reinforced mortar

## Abstract

Fabric-reinforced cementitious matrix (FRCM) composites are strengthening systems composed of a technical textile embedded in a cementitious or lime-based matrix and are increasingly used for strengthening existing masonry and concrete structures due to their compatibility with traditional substrates. The mechanical behavior of FRCM composites is controlled by the combined contribution of the textile reinforcement, the matrix, and the interface developed between them, with the textile–matrix interface playing a critical role in stress transfer, crack development, and post-cracking response. Since this interface is primarily defined by the coating applied to the textile, coating configuration represents a key parameter influencing both the mechanical and durability performance of the composite. In this study, carbon textile–reinforced FRCM systems incorporating a lime-based matrix and different coating strategies, including single-layer SBR coatings and multilayer SBR–epoxy coatings, were experimentally investigated. Tensile tests were conducted on unconditioned specimens as well as after exposure to water and alkaline environments to assess the evolution of tensile behavior and damage mechanisms under durability-related conditioning. The results indicated that the influence of coating configuration is slightly detectable in the pre-cracking elastic stage but becomes significant in the post-cracking stages, where load transfer and damage evolution are predominantly governed by the textile–matrix interface. Scanning electron microscopy (SEM) observations supported the mechanical findings by revealing distinct differences in coating, interfacial continuity, and fiber–matrix bonding, particularly after environmental exposure. Overall, the multilayer coating configuration, consisting of the factory SBR-coated carbon textile further modified with epoxy, resulted in higher maximum tensile strength (reaching up to 1958 MPa compared with 1531–1780 MPa for the single SBR-coated configuration), greater strain capacity (ε_max_ up to 0.01244 mm/mm compared with 0.00925–0.01066 mm/mm), and higher energy absorption under prolonged water and alkaline conditioning up to 3000 h. In quantitative terms, the multilayer SBR–epoxy coating improved the maximum tensile stress by approximately 10–15% and the total energy absorption capacity by 25–35%, depending on the conditioning regime. These findings demonstrate the effectiveness of multilayer coating architecture in improving long-term tensile retention, interfacial stress transfer, and post-cracking deformation capacity of lime-based carbon FRCM systems.

## 1. Introduction

In recent years, Fabric Reinforced Cementitious Matrix (FRCM) systems have gained increasing attention as an effective solution for the strengthening and rehabilitation of existing structures, particularly in the context of architectural heritage [1,2,3,4,5]. In European practice, these systems are commonly referred to as Textile Reinforced Mortars (TRM), whereas American guidelines adopt the term FRCM; despite the difference in terminology, both describe the same class of inorganic matrix–based composite systems [6,7]. The FRCM system consists of a cement or mineral-based mortar matrix combined with an open-mesh textile reinforcement. The mortar matrix governs crack initiation and stress redistribution while protecting the fibers, whereas the textile primarily controls tensile resistance and post-cracking behavior [8,9,10]. When compared to Fiber Reinforced Polymer (FRP) composites, FRCM composites are reported to offer several advantages in terms of their application in buildings and are therefore preferred in many applications. Compared with conventional FRP composites, FRCM systems offer advantages that are especially relevant for historic constructions, including improved compatibility with masonry substrates, vapor permeability, reversibility, and superior fire resistance [11,12,13]. Lime-based mortars were preferred because of their chemical and mechanical compatibility with historic masonry substrates and their high vapor permeability. Their relatively low elastic modulus and mechanical stiffness also help minimize incompatibility stresses and reduce the risk of irreversible intervention during strengthening applications [14,15,16]. These characteristics align with established conservation principles and international charters, which emphasize material compatibility and minimal intervention [17]. While FRP systems experience a rapid reduction in mechanical properties at relatively low temperatures, FRCM composites retain stable performance at significantly higher temperatures, making them more consistent with conservation requirements and international recommendations [8,18,19].

An FRCM system consists of multiple phases, including the interfaces formed as a result of applying various coatings onto the continuous fibers in order to facilitate handling and application. The characteristics of the phases within the system can significantly influence the overall properties of the composite. The overall mechanical response of FRCM composites is defined not only by the individual constituents, but also by the efficiency of stress transfer at the textile–matrix interface [20,21,22].

The type of textile reinforcement plays a decisive role in the mechanical and durability performance of FRCM systems. Glass and basalt fibers are widely used due to their economic advantages, yet numerous studies have shown that they are susceptible to degradation in alkaline environments [23,24,25]. Glass and basalt fibers are also questioned in terms of their use in reinforced concrete structures, particularly due to their relatively low elastic moduli. Polyparaphenylene benzobisoxazole (PBO) fibers offer very high tensile strength but have demonstrated limited durability under sustained exposure conditions [26,27,28]. Carbon fibers, in contrast, combine high tensile strength, elevated elastic modulus, and superior chemical stability, making them particularly suitable for long-term strengthening applications [29,30]. For these reasons, carbon textiles are increasingly selected for lime-based FRCM systems when higher load capacity and durability are required [31].

Although fiber type contributes to composite performance, the fiber–matrix interface is widely regarded as the dominant factor governing the overall mechanical response. This interface controls load transfer efficiency and bond–slip behavior within the composite system. Moreover, the interfacial performance is highly dependent on the type of coating applied to the fibers. To enhance bond efficiency and protect fibers from environmental attack, polymeric surface coatings are commonly applied to textile reinforcements [32,33]. Epoxy coatings are known to provide strong adhesion and high initial bond strength; however, their inherent rigidity and susceptibility to cracking under long-term exposure may influence durability [34,35]. Styrene–butadiene rubber (SBR) coatings, on the other hand, are flexible polymer films that promote cohesion, crack distribution, and deformability [36,37]. Nevertheless, previous studies indicate that SBR coatings may undergo changes under prolonged exposure to moisture or alkaline environments, affecting bond retention over time [38,39]. As a result, the long-term behavior of FRCM systems is closely related to the stability of the coating–matrix interface [40].

Most experimental studies addressing coated textiles in FRCM systems have focused on single-layer coating strategies, in which one polymer coating is applied to modify the textile–matrix interface [41,42]. These approaches have provided valuable insights into bond mechanisms and mechanical response; however, they represent only one level of interfacial modification. In practical applications, carbon textiles are often supplied with a factory-applied SBR coating, designed to improve handling and crack distribution, and this primary coating may be supplemented by an additional polymer layer, such as epoxy, applied during installation [43]. The introduction of a multilayer coating modifies the interfacial architecture of the composite system, potentially affecting its mechanical and durability performance. In practical FRCM applications, this multilayer strategy is particularly relevant because commercially available carbon textiles are most commonly supplied with factory-applied SBR precoating, which serves as the standard industrial baseline. The present multilayer concept was therefore motivated by the hypothesis that combining the inherent ductility and crack redistribution capacity of SBR with the higher stiffness and barrier effect of an additional epoxy overlayer may provide a more balanced tensile response together with improved durability stability [32,41].

The multilayer reinforcement concept investigated in this study was not primarily developed from an economic-efficiency perspective, but rather for strategic strengthening scenarios in which structural reliability and collapse prevention are the governing design priorities. The intended application domain includes critical infrastructure and strategically important wall systems, such as containment-type structural walls where prevention of in-plane collapse and defect-sensitive crack propagation is a primary safety requirement. In such cases, the additional coating phase is justified by the need for enhanced durability retention and stable post-cracking tensile behavior, even if the material system becomes more complex than conventional strengthening solutions.

From a mechanical properties perspective, the introduction of an additional coating layer has the potential to modify stress-transfer mechanisms at multiple length scales, influencing fiber interaction, crack spacing, stiffness evolution, and post-cracking response [44]. From a durability standpoint, the coexistence of polymer layers with different stiffness, permeability, and chemical resistance may affect the progression of interfacial degradation under prolonged exposure to water or alkaline environments [38,45]. Previous durability studies on FRCM systems have shown that environmental actions should be evaluated considering not only the reinforcement itself, but also the mortar matrix and the textile–matrix interface, which collectively govern residual tensile retention under long-term conditioning. In alkaline durability protocols commonly adopted for FRCM systems, the exposure medium is generally defined as a highly alkaline pore-like environment with pH levels above 12 and laboratory temperatures typically maintained at 20–25 °C, enabling a quantitative comparison of conditioning severity across different studies. Under such conditions, degradation of the mortar phase and the progressive weakening of the interfacial stress-transfer zone may become more critical than the intrinsic chemical stability of carbon fibers alone. Therefore, the durability of the coating architecture becomes particularly relevant for long-term performance [26,46,47,48]. These effects are particularly relevant for lime-based FRCM systems, where both the mortar matrix and the coating layers are more sensitive to environmental actions [49]. A detailed examination of layered coating architectures may therefore contribute to a more comprehensive understanding of long-term FRCM performance [50].

Within this context, the present study investigates lime-based FRCM composites reinforced with commercially available SBR-precoated carbon textiles, with and without an additional epoxy multilayer coating. Specimens were subjected to prolonged conditioning in water and alkaline solutions for durations of up to 3000 h, following commonly adopted durability protocols [51]. The mechanical behavior of the composite specimens was evaluated through direct tensile testing under different conditioning states. The mortar matrix was characterized through flexural and compressive tests according to EN 1015-11 [24,29,52]. By focusing on the role of an additional multilayer coating, the study aims to clarify how multilayer polymer coatings influence stress transfer, bond retention, and tensile performance of carbon textile–reinforced lime-based FRCM systems under extended environmental exposure.

## 2. Materials and Test Methods

The experimental program was designed as a controlled parametric investigation to evaluate the performance of FRCM composites incorporating textiles with a multilayer coating configuration. A single carbon textile reinforcement type was intentionally selected in order to isolate the influence of coating architecture from the intrinsic effects of fiber type. Carbon technical textiles already coated with SBR were further modified by applying an additional epoxy layer to obtain the multilayer coating system. The experimental variables were limited to the coating configuration, conditioning environment, and exposure duration, while all other parameters, including textile architecture, mortar composition, specimen geometry, curing conditions, and tensile testing setup, were kept constant. The coated textiles were embedded within a lime-based mortar matrix to produce FRCM composite specimens under controlled laboratory conditions. After curing, the specimens were categorized into unconditioned and durability-conditioned groups. Durability exposure was conducted under controlled water and alkaline environments for predefined durations (1000, 2000, and 3000 h) to simulate aggressive service conditions, following the environmental exposure principles of EAD 340275-00-0104 together with the coupon-level durability qualification logic of AC434, adapted for lime-based carbon textile FRCM specimens. Following the conditioning phase, all specimens were subjected to direct tensile testing according to RILEM 232 TDT. Following the mechanical tests, selected specimens were examined using scanning electron microscopy (SEM) to investigate fracture surfaces, coating integrity, and fiber–matrix interaction mechanisms. The experimental framework ensured consistent fabrication, curing, conditioning, and testing procedures across all specimen series.

### 2.1. Materials

#### 2.1.1. Textile Reinforcement

**Figure 1:**
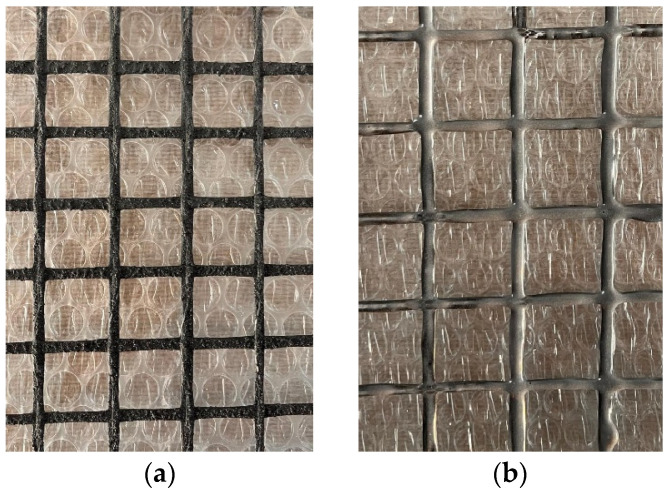
(**a**) SBR-coated carbon textile grid, (**b**) multilayer-coated (SBR + Epoxy) carbon textile grid.

A single type of carbon textile grid reinforcement was used to produce the FRCM composites. The textile was commercially supplied in an SBR-precoated form, and all material properties were reported according to the manufacturer’s declaration. The textile had an areal density of 370 g/m^2^, a grid opening of 25 × 25 mm, a density of 1.78 g/cm^3^, a single-fiber tensile strength of 4300 MPa, a thickness of 1.2 mm, a cross-sectional area of 71 mm^2^/m, an elastic modulus of 240 GPa, and an elongation at break of 1.8%. These properties are summarized in Table 1. Coated carbon textile grids are shown in Figure 1.

#### 2.1.2. Mortar Matrix

A hydraulic lime–based mortar was used to produce the FRCM composites. Lime-based mortars were preferred because of their compatibility with historical masonry substrates, breathability, low elastic modulus and low mechanical strength, which reduces incompatibility and irreversible interventions during strengthening applications.

The commercial mortar contained natural hydraulic lime, siliceous sand, a polycarboxylate-based superplasticizer, and water. NHL was selected as a binder due to its breathability and compatibility with masonry substrates. The aggregate was a washed siliceous river sand with 0–2 mm particle size, compliant with EN 13139. A polycarboxylate-based superplasticizer, corresponding to ≈1% of the binder weight, was added to enhance workability, and the water content was adjusted to a water-to-binder ratio of 0.5 to ensure consistent flow. Mortar properties were presented in Table 2. In addition, the 28-day mechanical properties of the mortar matrix were determined according to EN 1015-11 using 40 × 40 × 160 mm prism specimens. The average flexural and compressive strengths were measured as 1.73 MPa and 9.12 MPa, respectively. The mortar was supplied as a commercially premixed hydraulic lime-based strengthening mortar. During specimen preparation, the dry premixed mortar was first homogenized, after which the required amount of water and the polycarboxylate-based superplasticizer were gradually introduced under controlled mechanical mixing. Mixing was performed using a laboratory mixer at 600 rpm until a uniform and workable consistency was achieved before casting into the molds.

#### 2.1.3. Coating Materials

##### SBR

The carbon textile grid reinforcements used in this study were supplied with a factory-applied styrene–butadiene rubber (SBR) coating. The coating was applied by the manufacturer prior to composite fabrication to improve cohesion, handling properties, and fiber–matrix interaction. The polymer dispersion has a solids content of 47%, a pH value of 9.3, and a low viscosity (<300 mPa·s), allowing uniform impregnation of the textile fibers. The SBR coating content and application conditions were kept constant for all SBR-coated textiles, and no additional surface treatment was applied prior to specimen fabrication. The SBR coating does not form a chemical bond with the cementitious matrix; its contribution is primarily physical, enhancing fiber impregnation and stress transfer at the fiber–matrix interface. The coating configuration was identical for all SBR specimens; therefore, any differences in performance are attributed to the applied conditioning regimes.

##### Epoxy Coating

An epoxy-based coating was applied to the carbon textiles used in this study. A commercially available two-component epoxy resin system (EPX-FRP) was selected and prepared by mixing the resin and hardener immediately prior to application in accordance with the manufacturer’s recommendations. The epoxy coating was applied manually by brushing onto the as-received carbon textiles on both sides to ensure effective impregnation of the fibers while avoiding excessive resin accumulation. The effectiveness of the additional brushing process was quantified through areal mass measurements. The nominal areal density of the textile increased from 370 g/m^2^ in the factory SBR-coated state to approximately 500 g/m^2^ after epoxy overcoating. This corresponds to an added epoxy amount of about 130 g/m^2^, equivalent to an approximate mass increase of 35% relative to the initial SBR-coated textile. The viscosity of the epoxy at 23 °C (500 ± 25 mPa·s) enabled controlled application and uniform coating distribution. After application, the epoxy-coated textiles were cured at ambient laboratory conditions. The epoxy system exhibits a pot life of approximately 180 ± 60 min, a touch-dry time of 24 h, and full curing after 7 days at 23 °C and 55% relative humidity. During curing, a rigid, continuous polymer network forms around the fibers. The cured epoxy coating is characterized by a density of 1.22 g/cm^3^, a Shore-D hardness of 89, tensile and flexural strengths exceeding 65 MPa and 100 MPa, respectively. After curing, the epoxy-coated textiles were used to fabricate specimens without any additional surface treatment.

### 2.2. Specimen Preparation

**Figure 2:**
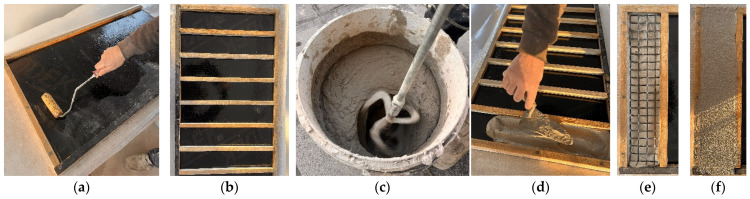
Fabrication stages of the FRCM composite specimens: (**a**) oiling of the mold surfaces, (**b**) preparation of plywood molds, (**c**) mechanical mixing of the cementitious mortar, (**d**) placement of the first mortar layer, (**e**) positioning of the textile on fresh mortar, and (**f**) final appearance of the composite specimen before curing.

FRCM composite specimens were prepared using plywood molds. The cementitious mortar was mixed using a mechanical mixer at a constant 600 rpm for the manufacturer-recommended duration to ensure homogeneity. Prior to casting, the technical textiles were cut to dimensions compatible with the plywood molds, ensuring full coverage of the specimen length and width while preserving the original fiber orientation and mesh geometry. The composites were fabricated in rectangular molds with internal dimensions of 500 mm × 100 mm × 20 mm. A thin layer of fresh mortar was first placed into the mold and leveled, after which the pre-cut textile was positioned onto the fresh mortar and gently pressed to promote impregnation and adequate bonding. Subsequently, a second mortar layer was applied to fully embed the textile, resulting in a total specimen thickness of approximately 20 mm, and the surface was leveled using a steel trowel. After casting, the specimens were covered to prevent premature moisture loss and cured under laboratory conditions for 28 days. The main fabrication stages of the FRCM composite specimens are illustrated in Figure 2.

#### 2.2.1. Durability Conditioning

After 28 days of curing under laboratory conditions, the FRCM composite specimens were subjected to durability conditioning to evaluate the influence of environmental exposure on their mechanical performance. Two different conditioning regimes were considered: water immersion and alkaline exposure. Unconditioned specimens were retained as reference samples. For water conditioning, the specimens were fully immersed in water at laboratory temperature. Complete submersion was maintained to ensure uniform exposure of the cementitious matrix and the embedded textile. Water immersion was applied for exposure durations of 1000, 2000, and 3000 h. For alkaline conditioning, the specimens were immersed in a lime-based alkaline solution containing 0.16% Ca(OH)_2_ by weight, representative of the alkaline environment typically encountered in cementitious matrices. The exposure temperature was maintained at 23 °C throughout the conditioning period. The specimens were fully submerged in the alkaline solution for 1000, 2000, and 3000 h, ensuring continuous contact between the solution, the mortar matrix, and the textile reinforcement. During alkaline conditioning, temperature, pH, and exposure time were monitored and maintained throughout the exposure period. The pH of the alkaline solution remained within the range of 12.5–12.6 throughout the entire conditioning period. Upon completion of the specified exposure durations, the specimens were removed from the conditioning media. Alkaline-conditioned specimens were gently rinsed with water to remove surface residues and surface-dried. Subsequently, all conditioned specimens were stored under laboratory conditions for 168 h to allow drying prior to tensile testing.

#### 2.2.2. Specimen Identification and Naming

A systematic nomenclature was adopted to clearly identify the textile coating type and the durability conditioning applied to each FRCM composite specimen. The specimen designation consists of a combination of textile coating type, conditioning environment, and conditioning duration. Textiles coated with styrene–butadiene rubber were denoted as SBR, while epoxy-coated textiles were denoted as EPX. Unconditioned reference specimens were identified by the suffix UC (e.g., SBRUC and EPXUC). Specimens subjected to durability conditioning were labeled using a letter indicating the exposure environment, followed by a numerical suffix representing the conditioning duration in hours. Specimens exposed to alkaline conditioning were denoted by the letter A, while those subjected to water immersion were denoted by the letter W. For example, SBRA1000 refers to an SBR-coated textile composite subjected to alkaline exposure for 1000 h, whereas EPXW1000 denotes an epoxy-coated textile composite exposed to water immersion for 1000 h. The same naming convention was applied for all exposure durations (1000, 2000, and 3000 h) and specimen groups.

- Test Methods
- Tensile Test

**Figure 3:**
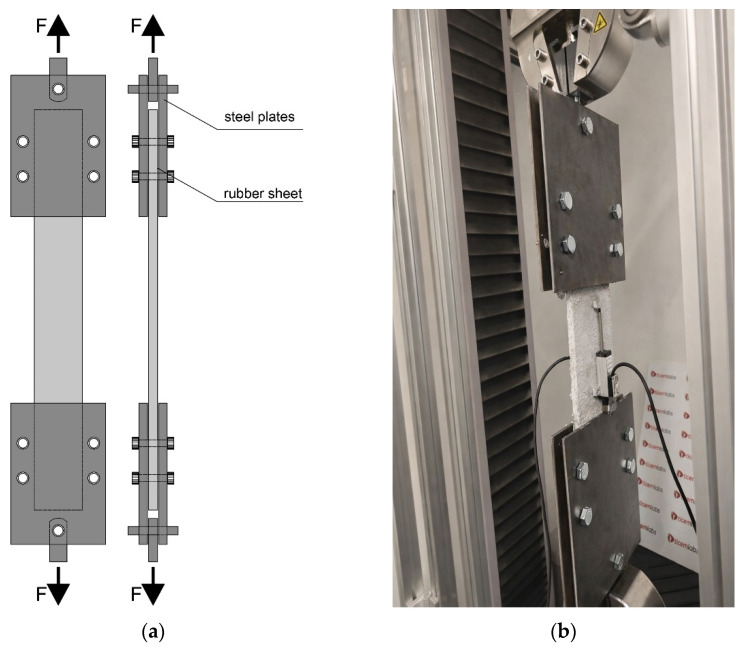
Tensile test setup for FRCM specimens: (**a**) schematic illustration of the clamping grid system showing steel plates and interposed rubber sheets; (**b**) experimental setup.

Tensile tests were performed on the FRCM composite specimens using a clamping grid system, in accordance with RILEM TC 232 TDT Recommendation for tensile characterization of FRCM systems. The clamping device consisted of steel plates with interposed rubber sheets to ensure uniform stress transfer and to prevent premature damage at the gripping zones. The load was applied axially through the clamping grids, as schematically illustrated in Figure 3.

Tensile tests were conducted using an MTS Criterion C43 504E universal servo-controlled testing machine with a maximum load capacity of 50 kN. The tests were performed under deformation-controlled loading, allowing stable crack development and post-cracking response to be captured. The laboratory temperature during testing was maintained at 18 ± 2 °C. Axial deformation was measured using an extensometer mounted on the specimen gauge length, enabling direct strain measurement in the composite’s central region. This configuration minimized the influence of grip compliance and ensured accurate evaluation of the tensile stress–strain response. All tensile tests were carried out following the same setup and loading protocol to ensure consistency and comparability among the different specimen groups. For each test configuration, five nominally identical specimens were tested, and the reported results represent the mean values with the corresponding standard deviations.

**Figure 4:**
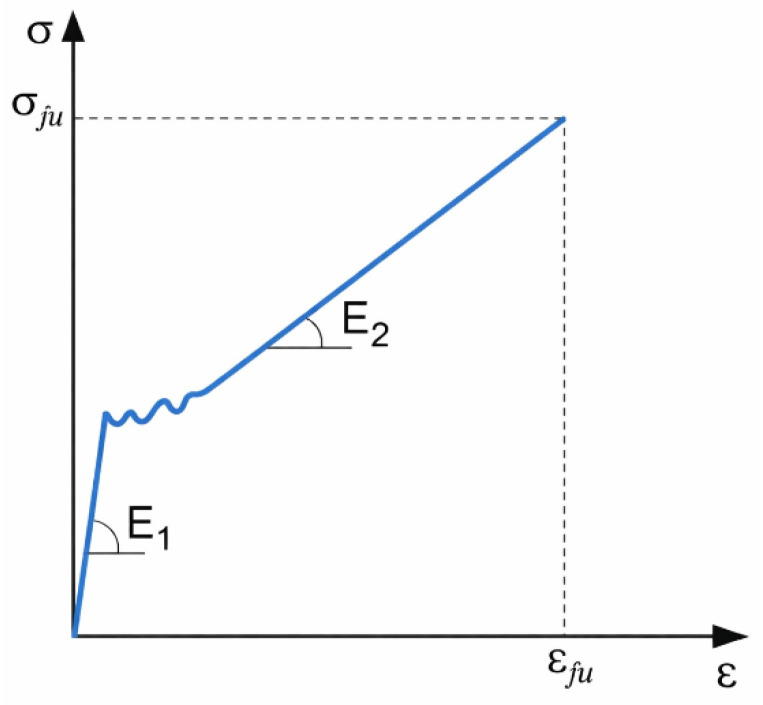
Schematic representation of the tensile stress–strain response of FRCM composites showing the uncracked stage (E_1_), post-cracking stage (E_2_), and ultimate tensile stress (σ_fu_) with corresponding strain (ε_fu_).

The typical tensile stress–strain response of FRCM composites is characterized by three distinct stages: an initial uncracked stage, a crack development stage, and a fully cracked stage, illustrated in Figure 4. In the uncracked stage, the composite response is governed by the elastic behavior of the matrix, and the corresponding stiffness E_1_ is defined as the slope of the stress–strain curve in this region. As the applied load increases, matrix cracking initiates and progressively develops, leading to a transition toward the cracked stage, where load transfer is primarily controlled by the textile reinforcement and the fiber–matrix interaction. The stiffness in this stage, denoted as E_2_, is determined from the slope of the stress–strain curve in the post-cracking region. The ultimate tensile strength, σ_fu_, corresponds to the maximum stress attained during the tensile test, while the associated strain, ε_fu_, represents the ultimate tensile strain of the composite.

This schematic representation provides a consistent framework for defining and comparing tensile parameters derived from the experimental stress–strain curves analyzed in this study. In the tensile evaluation, the stress was calculated by dividing the applied load by the effective cross-sectional area of the fibers aligned in the loading direction. The composite cross-sectional area was not used for stress calculation. This approach was adopted because, in FRCM strengthening applications, once matrix cracking occurs, the tensile load is primarily carried by the textile grid reinforcement. Therefore, the mechanical performance of the system is governed by the stress developed in the fibers rather than by the overall composite section. Accordingly, all stress–strain curves presented in this study represent fiber-based stresses derived from the experimental load data.

### 2.3. SEM Analysis

SEM was employed to investigate the microstructural features and damage mechanisms of the FRCM composite specimens after tensile testing. SEM observations were performed using a Thermo Scientific Phenom ProXL scanning electron microscope. Selected specimens representing different textile coating types (SBR and EPX) and durability conditions (unconditioned, water-immersed, and alkaline-exposed) were examined. Representative fragments were extracted from the gauge region of the specimens after tensile testing. Prior to SEM observation, the samples were coated with a Pt/Au conductive layer to reduce charging and enhance image quality. SEM imaging was conducted under high-vacuum conditions, with an accelerating voltage of 15 kV. The fractured surfaces were analyzed to evaluate the fiber–matrix interface, fiber impregnation quality, matrix cracking, and failure mechanisms, including fiber rupture, pull-out, and interfacial debonding. Particular attention was given to comparisons between unconditioned specimens and those subjected to long-term water and alkaline exposure to identify durability-induced microstructural changes. SEM analysis was used as a complementary tool to correlate the observed microstructural features with the tensile stress–strain response and mechanical performance obtained from macroscopic testing.

## 3. Test Results

### 3.1. Tensile Test Behavior of FRCM Composites

**Figure 5:**
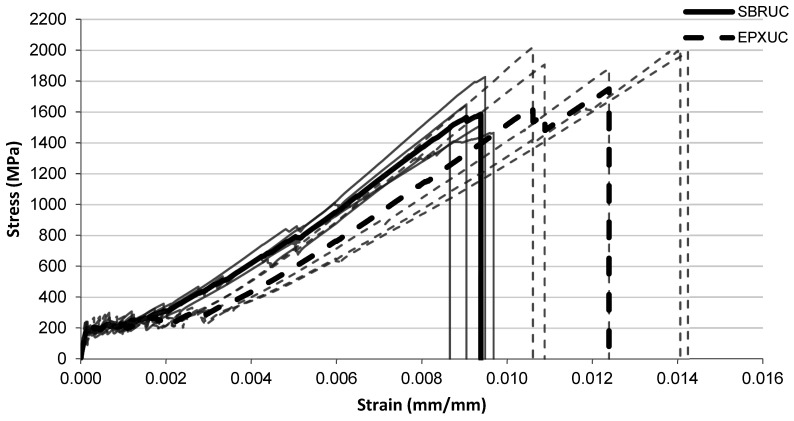
Tensile stress–strain relationship of FRCM specimens with SBRUC SBR-coated and EPXUC multilayer coated systems.

**Figure 6:**
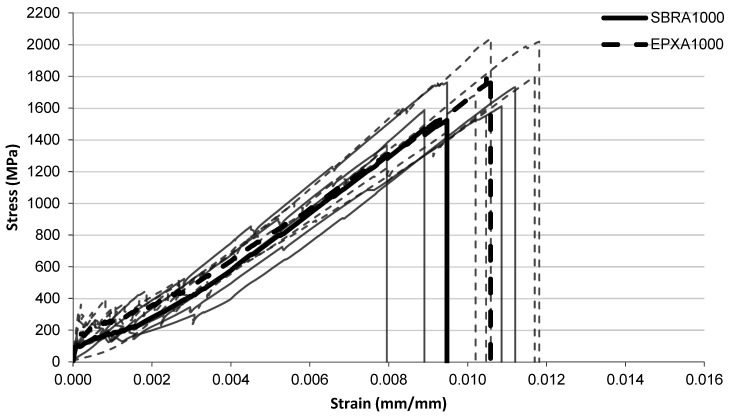
Tensile stress–strain relationship of FRCM specimens after alkaline conditioning (1000 h) with SBRA1000 and EPXA1000 multilayer coated systems.

**Figure 7:**
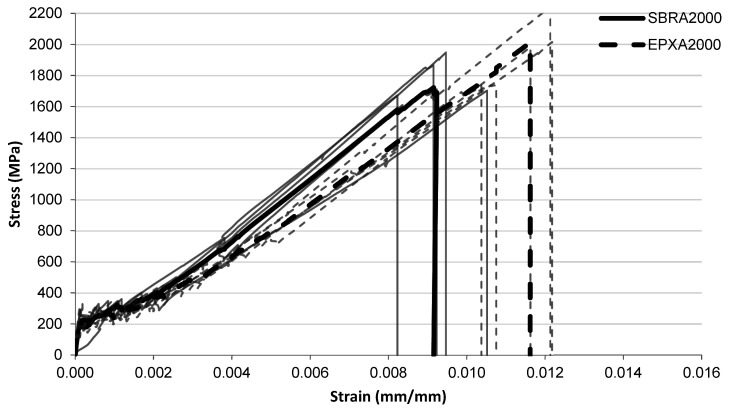
Tensile stress–strain relationship of FRCM specimens after alkaline conditioning (2000 h) with SBRA2000 and EPXA2000 multilayer coated systems.

**Figure 8:**
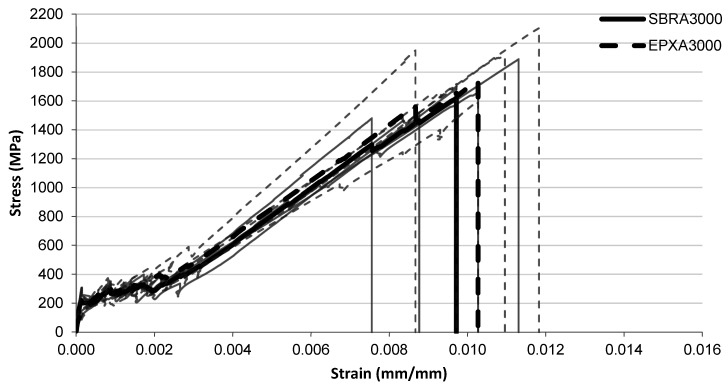
Tensile stress–strain relationship of FRCM specimens after alkaline conditioning (3000 h) with SBRA3000 and EPXA3000 multilayer coated systems.

**Figure 9:**
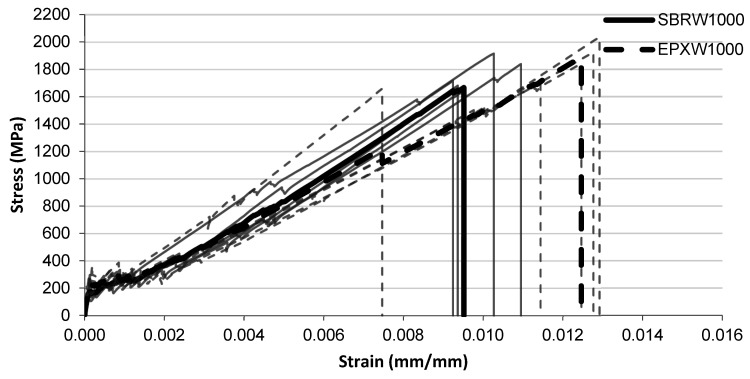
Tensile stress–strain relationship of FRCM specimens after water immersion (1000 h) with SBRW1000 and EPXW1000 multilayer coated systems.

**Figure 10:**
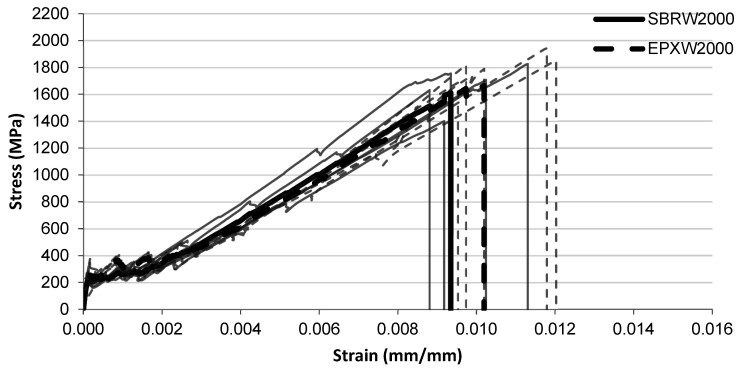
Tensile stress–strain relationship of FRCM specimens after water immersion (2000 h) with SBRW2000 and EPXW2000 multilayer coated systems.

**Figure 11:**
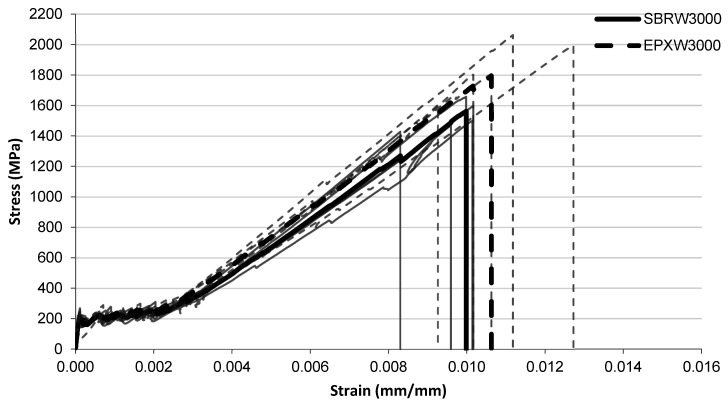
Tensile stress–strain relationship of FRCM specimens after water immersion (3000 h) with SBRW3000 and EPXW3000 multilayer coated systems.

The tensile stress–strain responses of the investigated FRCM composites are presented in Figure 5, Figure 6, Figure 7, Figure 8, Figure 9, Figure 10 and Figure 11 for different conditioning states. For each configuration, the responses of five individual specimens are shown together with the corresponding mean curves.

All specimens exhibited the typical three-stage tensile behavior commonly observed in FRCM systems. The first stage corresponds to the uncracked elastic response governed by the mortar matrix. This stage is followed by the crack development phase, during which multiple cracks progressively form along the specimen length. After crack stabilization, the tensile response becomes primarily governed by the textile reinforcement, resulting in the post-cracking stage characterized by reduced stiffness and increasing strain capacity.

#### 3.1.1. Unconditioned Specimens

The tensile stress–strain responses of the unconditioned specimens are presented in Figure 5. The initial portions of the curves for the SBRUC and EPXUC systems exhibit very similar behaviour, indicating comparable stiffness at low strain levels. As the strain increases, both systems show a similar stress development up to stress levels of approximately 1400–1600 MPa. However, differences become more visible in the final deformation stage. The SBRUC specimens generally fail at strain levels around approximately 0.009–0.0095, whereas the EPXUC specimens reach higher strain levels before failure, typically in the range of approximately 0.011–0.014. In addition, several EPXUC curves extend to higher stress levels approaching 1800–2000 MPa. The SBRUC curves appear relatively clustered, while the EPXUC curves exhibit a wider spread in the final deformation region.

#### 3.1.2. Alkaline Conditioning

The tensile stress–strain responses of the specimens exposed to alkaline conditioning are presented in Figure 6, Figure 7 and Figure 8 for exposure durations of 1000 h, 2000 h, and 3000 h, respectively.

After 1000 h alkaline exposure (Figure 6), both coating systems exhibit similar stress development throughout most of the strain range. The SBR-A1000 specimens generally fail at strain values around 0.009–0.010, with stress levels close to 1500–1600 MPa. In contrast, the EPX-A1000 specimens reach slightly higher strain values before failure, typically around 0.010–0.012, and some curves approach stress levels close to 1800–2000 MPa. The SBR curves remain relatively clustered, while the EPX curves show a broader spread in the final deformation region. For the specimens conditioned for 2000 h (Figure 7), the initial portions of the curves again remain similar for the two coating systems. In the intermediate strain range, the SBR-A2000 curves appear slightly higher than the EPX-A2000 curves, indicating somewhat higher stress levels in this region. The SBR-A2000 specimens generally fail at strain levels around approximately 0.009, with stress levels close to 1650–1750 MPa. In contrast, the EPX-A2000 specimens reach larger strain values before failure, typically around 0.011–0.012, with several curves extending to stress levels approaching 1900–2100 MPa.

For the longest alkaline exposure duration of 3000 h (Figure 8), the stress development of the two systems becomes very similar throughout most of the strain range. The SBR-A3000 specimens typically fail at strain values around 0.0095–0.010, corresponding to stress levels close to 1600–1700 MPa. The EPX-A3000 specimens again reach slightly higher strain values before failure, generally around 0.0105–0.012, with some curves approaching stress levels close to 1700–2000 MPa. As observed in the previous conditions, the SBR curves remain relatively clustered, whereas the EPX curves exhibit a wider distribution in the final deformation region.

#### 3.1.3. Water Conditioning

The tensile stress–strain responses after water conditioning are presented in Figure 9, Figure 10 and Figure 11 for exposure durations of 1000 h, 2000 h, and 3000 h, respectively.

After 1000 h of water exposure (Figure 9), the initial portions of the curves remain similar for both coating systems. In the intermediate strain range, the SBRW1000 curves appear slightly higher than the EPXW1000 curves. The SBRW1000 specimens generally fail at strain levels around 0.009–0.010, corresponding to stress values close to 1600–1700 MPa. In comparison, the EPXW1000 specimens reach larger strain values before failure, typically around 0.012–0.013, with several curves approaching stress levels of approximately 1800–2000 MPa.

For the specimens conditioned for 2000 h in water (Figure 10), both systems show very similar stress development throughout most of the strain range. The SBRW2000 specimens generally fail at strain levels around 0.009–0.010, corresponding to stress values of approximately 1600–1700 MPa. The EPXW2000 specimens reach slightly higher strain levels before failure, typically around 0.010–0.011, with several curves approaching stress levels close to 1600–1800 MPa.

After the longest water exposure duration of 3000 h (Figure 11), the stress development of the two systems remains similar throughout most of the strain range. The SBRW3000 specimens typically fail at strain levels around 0.0095–0.010, corresponding to stress levels of approximately 1500–1600 MPa. In contrast, the EPXW3000 specimens again reach slightly higher strain values before failure, generally around 0.0105–0.011, with several curves approaching stress levels close to 1700–1800 MPa.

To provide a more direct quantitative comparison of the tensile behavior under alkaline exposure, the key parameters extracted from the stress–strain curves are summarized in Table 3. The stiffness parameters E1 and E2 were derived from the reconstructed mean curves to reduce the influence of local noise in the initial linear region, while the maximum tensile strength (σ_max_) and corresponding strain (ε_max_) are reported as specimen-based mean values together with their associated standard deviation and coefficient of variation (COV). This presentation allows the tensile capacity, deformation response, and variability of each conditioning series to be evaluated in a compact and consistent manner.

For a more compact evaluation of the tensile performance after water immersion, the main response parameters obtained from the stress–strain curves are presented in Table 4. In this summary, E1 and E2 represent the stiffness values derived from the reconstructed mean curves, ensuring a stable comparison of the linear and cracked response stages. In contrast, the maximum tensile strength (σ_max_) and the corresponding maximum strain (ε_max_) are given as specimen-based mean values together with their standard deviation and coefficient of variation (COV), which enables the influence of water exposure on both the peak response and result dispersion to be assessed more directly across all series.

### 3.2. Maximum Tensile Properties

To enable a quantitative comparison of the tensile performance of the investigated FRCM composites, the maximum tensile strength (σ_max_) and the corresponding strain at maximum (ε_max_) were extracted from the stress–strain curves for all test groups, considering the effects of coating type and exposure conditions (water immersion and alkaline exposure). The reported values represent the average of five specimens per group (*n* = 5), and the variability is expressed in terms of standard deviation.

**Figure 12:**
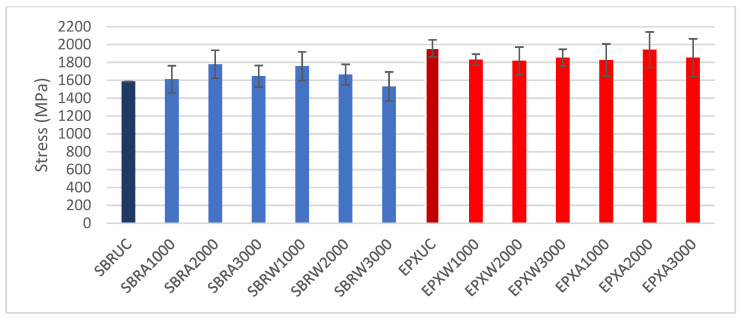
Maximum tensile strength (σ_max_) of SBR- and EPX-multilayer coated FRCM composites under unconditioned, water, and alkaline exposure conditions (mean ± SD, *n* = 5).

The variation in maximum tensile strength (σ_max_) for all investigated groups is presented in Figure 12. Under unconditioned (UC) conditions, the SBR-coated specimens exhibited maximum stress values of approximately 1580 MPa, while the EPX-coated specimens reached approximately 1750 MPa.

Following water immersion, the SBR series showed σ_max_ values ranging between approximately 1500 and 1780 MPa depending on the exposure duration. The EPX multilayer coated specimens under water conditioning exhibited peak stress values between 1800 and 1950 MPa.

After alkaline exposure, both coating systems demonstrated fluctuations in σ_max_ with increasing conditioning duration. The SBR-coated specimens exhibited peak stress values within the range of approximately 1500–1700 MPa, whereas the EPX multilayer-coated specimens showed values between approximately 1650 and 1900 MPa.

Expressed as relative improvement, the EPX multilayer-coated specimens showed a 10.2% higher maximum tensile strength than the SBR-coated reference on unconditioned specimens. After water conditioning, the corresponding gains were 4.2%, 9.3%, and 21.1% for 1000 h, 2000 h, and 3000 h, respectively. Under alkaline conditioning, the improvements were 13.3%, 9.1%, and 12.6% for the same exposure durations.

**Figure 13:**
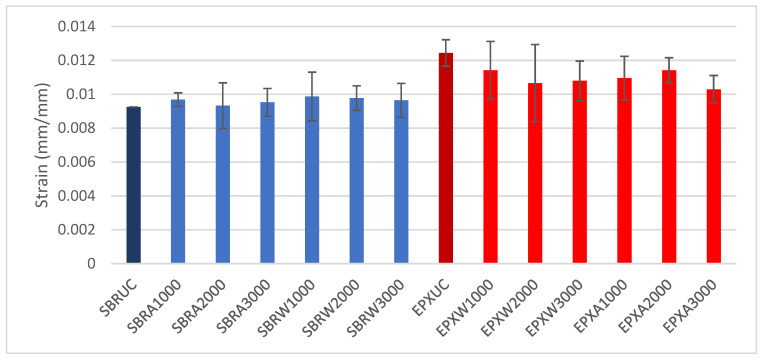
Maximum tensile strain (ε_max_) of SBR- and EPX-multilayer coated FRCM composites under unconditioned, water, and alkaline exposure conditions (mean ± SD, *n* = 5).

The maximum strain (ε_max_) values obtained for all groups are shown in Figure 13. The reported values represent the mean of five specimens per group (*n* = 5), and the variability is expressed in terms of standard deviation. The SBR-coated specimens exhibited relatively stable ε_max_ values across all conditioning states, remaining close to approximately 0.009–0.010 mm/mm.

In contrast, the EPX-coated specimens consistently reached higher strain levels, with ε_max_ values between approximately 0.010 and 0.012 mm/mm. Minor variations were observed with increasing exposure duration under both water and alkaline environments; however, the overall ε_max_ values of the EPX multilayer coated series remained higher than those of the SBR series throughout the investigated exposure conditions.

A similar trend was observed for the maximum strain. Compared with the SBR-coated reference, the EPX multilayer-coated specimens reached a 34.5% higher maximum strain on unconditioned specimens. Following water conditioning, the strain enhancement remained 15.6%, 9.0%, and 11.9% after 1000 h, 2000 h, and 3000 h, respectively. Under alkaline exposure, the corresponding increases were 13.2%, 22.4%, and 7.2% for the same conditioning durations. The more pronounced gains observed in ε_max_ compared with σ_max_ suggest that the multilayer coating primarily improves the post-cracking deformation capacity and the efficiency of interfacial stress redistribution rather than drastically altering the maximum tensile resistance.

### 3.3. Energy Absorption Capacity

**Figure 14:**
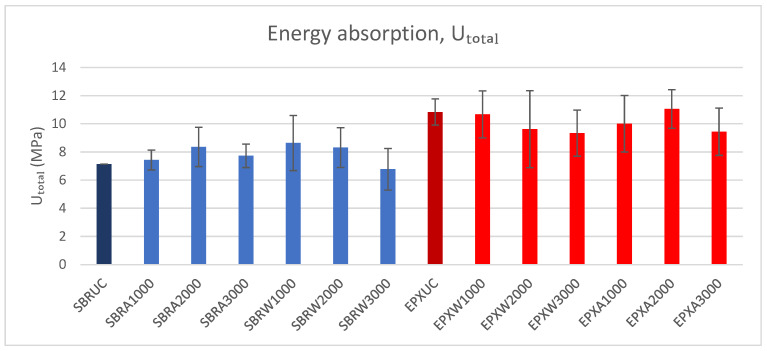
Energy absorption capacity (U_total_) of SBR- and EPX-multilayer coated FRCM composites under different exposure conditions (mean ± SD, *n* = 5).

The energy absorption capacity (U_total_) was calculated as the area under the tensile stress–strain curve up to failure. Since failure occurred immediately after peak stress for all specimens, U_total_ effectively corresponds to the integral of the stress–strain response up to ε_max_. The values represent the mean of five specimens per group (*n* = 5), and the variability is expressed in terms of standard deviation. The obtained U_total_ values are presented in Figure 14. It should be emphasized that the energy absorption capacity of FRCM composites is sensitive to the adopted tensile test setup, including the clamping configuration, gauge length, and deformation measurement method. Accordingly, the values reported here should be primarily interpreted as a comparative indicator between the investigated coating systems within the same experimental framework, while comparisons with absolute values from other studies should consider potential differences in test configuration.

To complement the graphical comparison shown in Figure 14, the mean energy absorption capacity values obtained from the tensile stress–strain curves are summarized in Table 5 together with the corresponding standard deviation and coefficient of variation (COV). This compact presentation enables a direct comparison of post-cracking deformation sustainability, result dispersion, and the influence of coating configuration under different environmental conditioning regimes.

The SBR-coated specimens exhibited energy absorption values ranging between approximately 7 and 9 MPa, depending on the conditioning duration. In contrast, the EPX multilayer coated specimens showed higher U_total_ values, typically between approximately 9 and 12 MPa across the investigated exposure conditions. Although moderate variations were observed with increasing conditioning duration, the multilayer-coated specimens consistently maintained higher energy absorption values than the SBR-coated series. Quantitatively, the EPX multilayer-coated specimens exhibited a 51.9% higher energy absorption capacity than the SBR-coated reference under unconditioned conditions. After water conditioning, the corresponding gains were 23.6%, 15.7%, and 37.9% for 1000 h, 2000 h, and 3000 h, respectively. Under alkaline conditioning, the increases were 34.7%, 32.2%, and 22.2% for the same exposure durations.

### 3.4. Microstructural Analysis (SEM)

Scanning electron microscopy (SEM) was performed to examine the post-failure fracture surfaces of the FRCM composites subjected to different exposure conditions. The analysis focused on (i) the morphology of fiber bundles, (ii) the distribution and adhesion of matrix residues, and (iii) the characteristics of the fiber–coating–matrix interfacial region. Images were acquired at two magnification levels (200 µm and 30 µm) to capture both global fracture morphology and localized interfacial features.

**Figure 15:**
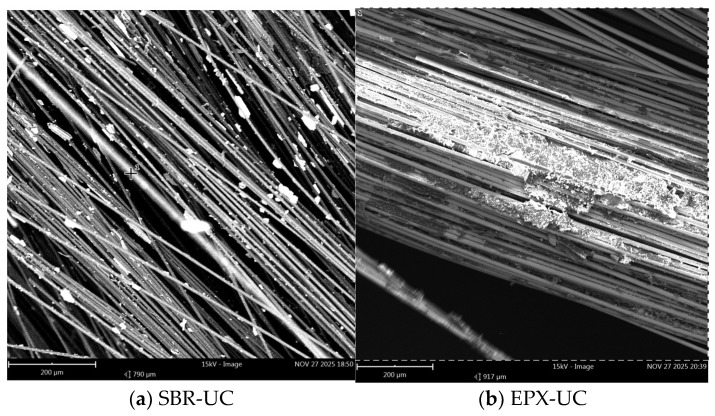
Low-magnification SEM images (200 µm) of unconditioned specimens: (**a**) SBR-coated FRCM; (**b**) EPX-multilayer coated FRCM.

At 200 µm magnification, the unconditioned SBR-coated specimen (Figure 15a) exhibited partially exposed fiber bundles with non-uniformly distributed mortar residues adhering to the fiber surfaces. Distinct bundle contours and localized separation zones between the bundles and surrounding matrix fragments were visible.

In contrast, the EPX-coated specimen under unconditioned conditions (Figure 15b) showed a comparatively more continuous peripheral layer surrounding the fiber bundles. Matrix remnants appeared more evenly distributed along the coated fibers, and fewer clearly isolated fiber segments were observed.

**Figure 16:**
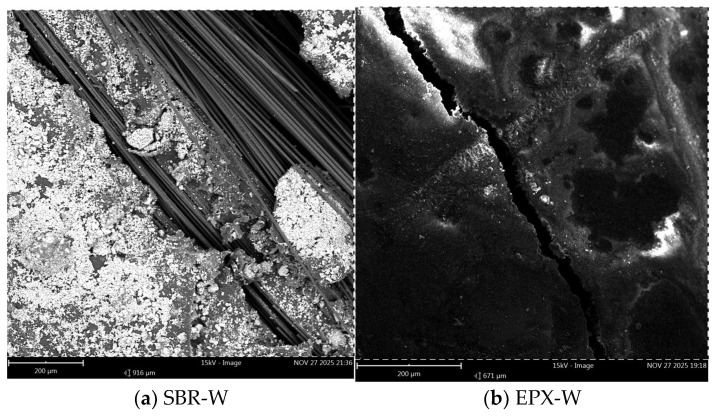
Low-magnification SEM images (200 µm) after water conditioning: (**a**) SBR-coated specimen; (**b**) EPX-multilayer coated specimen.

After water conditioning (Figure 16a,b), both coating systems demonstrated matrix fragmentation along the fracture surface. The SBR-coated specimen (Figure 16a) displayed visible bundle demarcation and discontinuous matrix attachment in several regions. The EPX multilayer coated specimen (Figure 16b) also exhibited fragmented mortar particles; however, localized areas of cohesive matrix attachment around portions of the bundles were more frequently observed.

**Figure 17:**
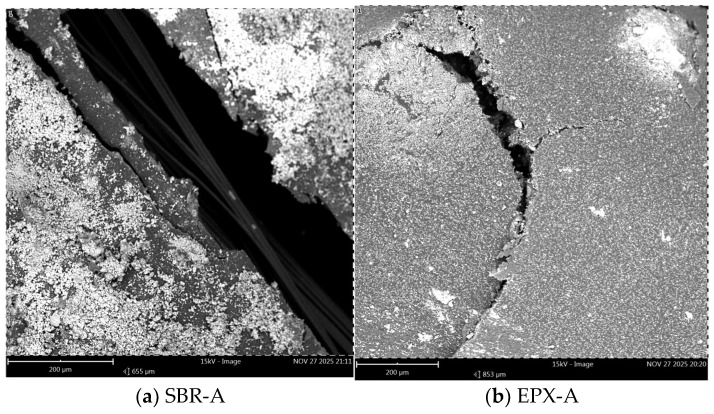
Low-magnification SEM images (200 µm) after alkaline conditioning: (**a**) SBR-coated specimen; (**b**) EPX-multilayer coated specimen.

Under alkaline exposure (Figure 17a,b), increased surface irregularity and matrix disintegration were evident in both systems. The SBR-coated specimen (Figure 17a) presented more pronounced bundle separation and detached matrix fragments, with clearly defined inter-bundle gaps in certain areas. The EPX multilayer coated specimen (Figure 17b) showed fragmented matrix particles as well; however, sections of the fracture surface still displayed relatively compact matrix coverage surrounding the fiber bundles.

**Figure 18:**
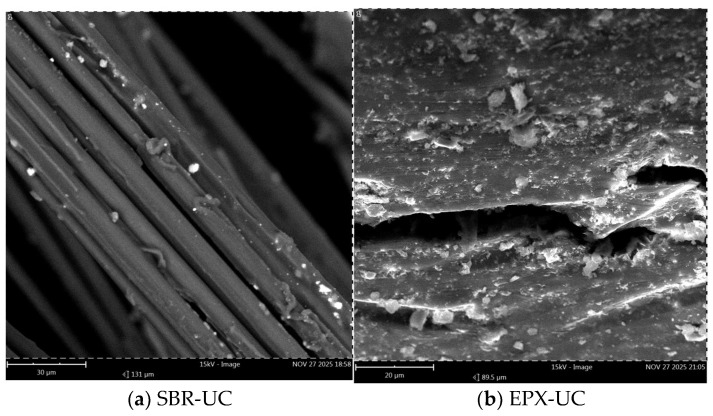
High-magnification SEM images (30 µm) of unconditioned specimens: (**a**) SBR-coated specimen; (**b**) EPX-multilayer coated specimen.

At higher magnification (30 µm), detailed features of the fiber–coating–matrix interface became apparent. The unconditioned SBR-coated specimen (Figure 18a) revealed partial exposure, localized coating thinning, and discontinuities along the fiber surface. Small interfacial void-like regions were observed at the boundary between the coating layer and the surrounding matrix.

In comparison, the unconditioned EPX multilayer coated specimen (Figure 18b) exhibited a comparatively more uniform coating thickness and a more continuous layer enveloping the fibers. The interfacial region appeared denser, and complete exposure was less frequently observed.

**Figure 19:**
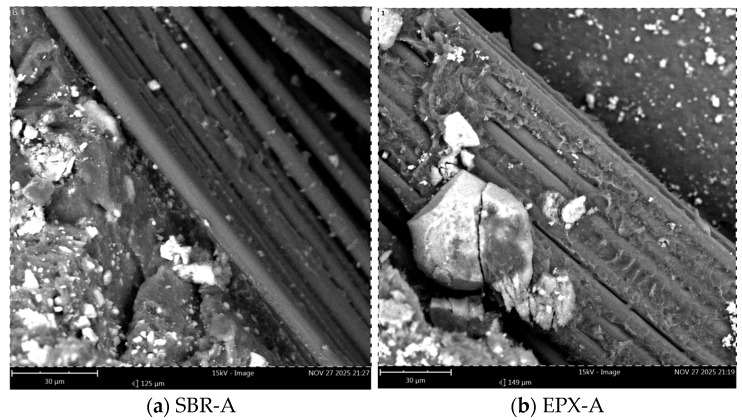
High-magnification SEM images (30 µm) after alkaline conditioning: (**a**) SBR-coated specimen; (**b**) EPX-multilayer coated specimen.

Following alkaline conditioning (Figure 19a,b), the SBR-coated specimen (Figure 19a) showed localized interfacial gaps and microcrack-like features propagating along portions of the fiber–matrix boundary. Regions of coating discontinuity and partial debonding were visible in selected areas.

The EPX multilayer coated specimen (Figure 19b) also displayed microcracks within the adjacent matrix; however, the coating layer surrounding the fiber bundles remained largely continuous, and extensive fiber exposure was not observed in the examined regions.

## 4. Discussion

The improved tensile response of the multilayer-coated specimens can be interpreted through a multiscale stress-transfer mechanism. The factory-applied SBR layer primarily promotes fibers and crack distribution because of its flexible nature, allowing local stress redistribution after matrix cracking. The additional epoxy overlayer creates a stiffer and more continuous interfacial shell around the textile bundles, which improves fiber engagement and reduces premature local debonding. After crack initiation in the mortar matrix, the tensile load is progressively transferred to the textile yarns through the coating-mediated interface. In the multilayer configuration, the stiffer epoxy shell enhances the uniformity of this transfer by mobilizing a larger number of fibers within the bundle and reducing local stress concentrations at partially bonded regions. As a result, stress transfer after crack formation becomes more uniform along adjacent yarns, delaying localized overload and promoting a more gradual post-cracking stiffness degradation. This interpretation is directly consistent with the experimentally observed increase in ultimate strain and total energy absorption of the EPX series under both water and alkaline conditioning. Therefore, the fracture mechanism interpretation in this study was established through the combined evaluation of the tensile stress–strain response, including post-cracking stiffness degradation, ultimate strain evolution, and energy absorption behavior, together with the post-failure SEM observations of fiber pull-out, matrix residue retention, and localized interfacial debonding. The experimental results demonstrate that the coating configuration plays a significant role in governing the tensile behaviour and durability performance of the investigated FRCM composites. Although both coating systems maintained their tensile capacity within the investigated exposure durations, clear differences emerged in terms of deformation behaviour and energy absorption. In particular, the specimens with SBR–epoxy multilayer coating exhibited higher deformation levels and higher energy absorption compared with the specimens coated only with SBR. These observations indicate that the coating configuration influences the efficiency of load transfer within the composite system, particularly at the textile–matrix interface, which plays a critical role in the tensile response of FRCM composites.

### 4.1. Influence of Coating Configuration on Tensile Behaviour

The comparison between the SBR-coated and SBR–epoxy multilayer coated specimens highlights the significant influence of coating configuration on the tensile behaviour of the investigated FRCM composites. The multilayer coating system exhibited higher deformation capacity and improved tensile performance compared with the specimens coated only with SBR. This behaviour can be attributed to the ability of the epoxy layer to enhance fiber impregnation and promote a more continuous coating around the textile yarns. Improved coating continuity may increase the efficiency of interfacial stress transfer between the textile reinforcement and the surrounding mortar matrix, particularly after matrix cracking, when the tensile response of the composite becomes governed by the textile phase [32,41].

In FRCM systems, the post-cracking stage is primarily controlled by the interaction between the textile reinforcement and the cementitious matrix. The presence of an additional epoxy layer may stabilize the fiber and reduce internal slippage among individual fibers, thereby allowing a more uniform stress distribution within the textile yarns. This mechanism may explain the higher deformation capacity and improved tensile response observed in the multilayer coated specimens.

From an interphase architecture perspective, the multilayer coating system can be interpreted as a hierarchical stress-transfer zone composed of an inner compliant SBR-rich phase and an outer stiffer epoxy-rich shell. The inner SBR layer maintains cohesion and accommodates local deformation incompatibilities during crack opening, whereas the outer epoxy layer enhances bundle confinement and promotes more effective load sharing among adjacent fibers. This layered interfacial architecture is particularly beneficial after matrix cracking, where the tensile load path becomes increasingly dependent on the continuity of the coating-mediated bond between the textile yarns and the surrounding mortar. Such a synergistic interaction between the two polymer layers provides a plausible intrinsic explanation for the sustained post-cracking deformation capacity and higher energy absorption observed in the multilayer-coated series.

The more pronounced improvements observed in ε_max_ and U_total_ compared with the relatively moderate gains in σ_max_ further suggest that the contribution of the multilayer coating is primarily related to composite-level stress redistribution rather than any change in the intrinsic tensile capacity of the carbon fibers. Based on the tensile response and SEM observations, the additional epoxy overlayer appears to improve the continuity of the coating around the fibers, which likely promotes more uniform stress transfer between the mortar matrix and the textile grid during crack propagation. This effect may reduce localized fiber slippage and delay stress concentration after matrix cracking, allowing the reinforcement to remain engaged over a larger deformation range. As a result, the multilayer-coated system exhibits a more sustained post-cracking tensile response, which is consistent with the higher ε_max_ and U_total_ values summarized in Table 3, Table 4 and Table 5.

Furthermore, the conditioning results indicate that the multilayer coating configuration provides an additional retention benefit under alkaline exposure. It should be considered that carbon textile-based FRCM systems are already known to exhibit relatively high resistance to alkaline environments compared with glass- or basalt-based counterparts. Therefore, the observed response should not be interpreted merely as a generic alkaline resistance effect of the carbon reinforcement itself. Rather, the multilayer-coated specimens demonstrated improved retention of tensile strength, deformation capacity, and energy absorption relative to the SBR-coated carbon reference, suggesting that the epoxy overlayer provides a measurable contribution beyond the intrinsic chemical stability of carbon fibers [51].

These results indicate that the multilayer coating enables a more efficient mechanical utilization of the textile reinforcement within the composite, leading to higher deformation capacity, improved energy absorption, and enhanced durability and stability compared with the single-layer SBR coating.

### 4.2. Effect of Alkaline Exposure on Coating Systems

Cementitious matrices are characterized by a highly alkaline pore solution, typically with a pH above 12, which can influence the durability of polymer coatings and the stability of the fiber–matrix interface in FRCM systems. Prolonged exposure to alkaline environments may alter the physicochemical stability of polymeric coatings and affect their adhesion properties and interfacial behaviour [33,36,43,53].

Although environmental conditioning is externally applied and therefore initially affects the mortar matrix, the retained tensile response after matrix cracking is primarily governed by the efficiency of stress transfer within the textile bundles. In this stage, the role of the coating architecture becomes critical because it controls confinement, local stress redistribution, and resistance to interfacial debonding. Therefore, the observed differences after water and alkaline exposure should be interpreted as matrix-mediated environmental ingress affecting the efficiency of textile utilization through the fiber–coating–matrix interphase, rather than as a direct change in the intrinsic properties of the carbon fibers. The moderate increases observed in selected SBR-conditioned groups are therefore interpreted as changes in crack redistribution and post-cracking textile engagement efficiency after conditioning and drying, rather than as a true intrinsic strengthening effect of the SBR coating itself.

In the present study, the SBR-coated specimens exhibited more noticeable variations in tensile behaviour after alkaline conditioning. Such variations may be associated with the interaction between the alkaline environment and the polymer phase surrounding the textile fibers. Changes in the polymer matrix may influence the efficiency of stress transfer between the textile reinforcement and the surrounding mortar, particularly during the post-cracking stage, where the load-carrying capacity is governed by the textile reinforcement.

In contrast, the specimens with SBR–epoxy multilayer coating exhibited improved retention of tensile response under alkaline conditioning relative to the SBR-coated carbon reference. Considering that carbon textiles are inherently less sensitive to alkaline exposure than glass- or basalt-based systems, the key outcome here is not the alkaline durability of the carbon fibers alone, but the additional contribution of the epoxy overlayer in preserving the fiber–matrix interfacial integrity. This barrier effect can reduce the penetration of the alkaline solution toward the fibers and the underlying SBR-coated interface, thereby contributing to the relatively stable mechanical performance observed in the multilayer-coated specimens. This protective effect is likely associated not only with reduced solution ingress but also with the preservation of the hierarchical interphase architecture established by the inner SBR and outer epoxy layers.

### 4.3. Microstructural Interpretation of Tensile Behaviour

The SEM observations provide insight into the microstructural mechanisms governing the tensile behaviour of the investigated FRCM composites. In textile-reinforced cementitious systems, the post-cracking tensile response is largely controlled by the efficiency of stress transfer between the textile reinforcement and the surrounding mortar matrix. Therefore, the integrity and continuity of the coating layer surrounding the fibers play an important role in determining the effectiveness of the fiber–matrix interaction.

Although full geometric encapsulation is not observed in every local region, the SEM images remain highly informative in a comparative sense by revealing relative differences in coating continuity and interfacial gap formation between the SBR and multilayer EPX systems.

The SEM images revealed clear differences in the morphology of the fiber bundles depending on the coating configuration. In the SBR-coated specimens, the fibers appeared relatively more exposed and locally separated from the surrounding mortar matrix (Figure 15a, Figure 16a and Figure 17a). The polymer coating surrounding the individual fibers appeared discontinuous in several regions, resulting in partially exposed fibers and irregular contact between the textile bundle and the matrix. Such discontinuities may reduce the efficiency of interfacial stress transfer and facilitate localized fiber slippage within the bundle during tensile loading.

In contrast, the multilayer coated specimens exhibited a more continuous polymer phase surrounding the textile yarns (Figure 15b, Figure 16b and Figure 17b). The epoxy overlayer appeared to form a relatively compact coating around the fibers, improving the confinement of the individual fibers. This microstructural configuration may enhance the mechanical coupling between the textile reinforcement and the surrounding mortar matrix, allowing a more uniform distribution of tensile strength within the yarn.

Higher-magnification observations further highlighted these differences. In the SBR-coated specimens, partially exposed fibers and local defects between the fibers and the surrounding matrix were observed (Figure 18a), suggesting a less continuous interfacial transition zone. Such features may promote interfacial debonding and localized fiber slippage during tensile loading. In contrast, the multilayer coated specimens exhibited a more uniform and continuous coating layer surrounding the fibers (Figure 18b), which may contribute to improved stress transfer within the reinforcement.

After alkaline conditioning, the differences between the two coating configurations became more evident. The SBR-coated specimens showed more pronounced separation between the fiber bundles and the surrounding matrix (Figure 17a and Figure 19a), indicating possible degradation of the interfacial region under alkaline exposure. Conversely, the multilayer coated specimens maintained a relatively continuous coating layer even after conditioning (Figure 17b and Figure 19b). This observation suggests that the epoxy overlayer may partially limit the penetration of the alkaline environment toward the textile reinforcement and the fiber–matrix interface.

The combined evaluation of the mechanical results and SEM observations highlights the critical role of coating architecture in controlling the tensile response and durability behaviour of FRCM composites. The multilayer SBR–epoxy coating provided improved confinement and enhanced interfacial stress transfer between the textile reinforcement and the surrounding mortar matrix. As a result, the multilayer-coated specimens exhibited higher deformation capacity, improved energy absorption, and improved retention of tensile response under environmental conditioning. Considering the already established chemical stability of carbon fibers in alkaline environments, these observations indicate that the multilayer coating strategy provides an additional interfacial retention benefit beyond the intrinsic durability of the carbon textile itself, which may contribute to the development of more reliable and durable FRCM strengthening systems for structural applications.

## 5. Conclusions

The present study investigated the influence of coating configuration on the mechanical and durability performance of carbon textile–reinforced lime-based FRCM composites incorporating single-layer SBR coatings and SBR–epoxy multilayer coatings. Based on the experimental results, the following conclusions can be drawn:

- The tensile behavior of the investigated FRCM composites exhibited the typical three-stage response of textile-reinforced cementitious systems, consisting of an uncracked stage governed by the mortar matrix, a crack development stage, and a fully cracked stage dominated by the textile reinforcement.
- Within the investigated conditioning durations, the EPX multilayer coating configuration generally resulted in higher tensile performance compared with the single-layer SBR coating. The maximum tensile strength of the multilayer-coated specimens reached values approaching approximately 1900–2000 MPa, whereas the SBR-coated specimens generally remained within the range of 1500–1700 MPa.
- The multilayer-coated specimens also exhibited greater deformation capacity, with maximum strain values typically between 0.010 and 0.012, compared with approximately 0.009–0.010 for the SBR-coated specimens.
- The energy absorption capacity of the multilayer-coated composites was higher across all exposure conditions, suggesting an improved ability of the composite to sustain deformation after matrix cracking and a more ductile tensile response.
- Durability conditioning revealed that alkaline exposure produced more noticeable variations in the tensile behavior of the SBR-coated specimens, whereas the multilayer-coated specimens showed improved retention of mechanical response under both water and alkaline environments.
- SEM observations indicated that the multilayer coating formed a more continuous polymer layer around the fibers, improving confinement and enhancing stress transfer efficiency between the textile reinforcement and the surrounding mortar matrix.
- The results highlight that the coating architecture of textile reinforcements represents an important design parameter in FRCM systems. Multilayer coating strategies may contribute to improved mechanical performance and durability retention in textile-reinforced cementitious composites, supporting the development of more reliable strengthening systems for existing structures. Future research may focus on FRCM composites produced with different textile types (e.g., glass or basalt fabrics) and alternative matrix systems in order to further evaluate the effectiveness of multilayer coating strategies under various environmental conditions.

## Figures and Tables

**Table 1:** Carbon textile physical properties.

Areal Density (gr/m^2^)	Grid Opening (mm × mm)	Density (g/cm^3^)	Thickness (mm)	Cross-Sectional Area (mm^2^/m)	Elongation at Break (%)
370	25 × 25	1.78	1.2	71	1.8

**Table 2:** Mortar properties.

Component	Amount (kg/m^3^)
Natural Hydraulic Lime (NHL)	600
Siliceous River Sand (0–2 mm)	900
Polycarboxylate Superplasticizer	6
Water	300

**Table 3:** Summary of tensile parameters of FRCM specimens subjected to alkaline conditioning.

Series	E1 (GPa)	E2 (GPa)	σ_max_ (MPa)	ε_max_ (mm/mm)
SBRUC	221.7	176.8	1587 ± 152 (9.6%)	0.00925 ± 0.00041 (4.4%)
SBRA1000	170.1	177.5	1612 ± 157 (9.7%)	0.00968 ± 0.00135 (14.0%)
SBRA2000	262.7	196.9	1780 ± 121 (6.8%)	0.00932 ± 0.00082 (8.8%)
SBRA3000	171.7	189.7	1646 ± 161 (9.8%)	0.00952 ± 0.00143 (15.3%)
EPXUC	260.5	180.2	1958 ± 62 (3.2%)	0.01244 ± 0.00171 (13.7%)
EPXA1000	290.9	167.3	1827 ± 200 (10.9%)	0.01096 ± 0.00075 (6.8%)
EPXA2000	260.4	176.6	1942 ± 212 (10.9%)	0.01141 ± 0.00082 (7.2%)
EPXA3000	325.1	191.9	1853 ± 203 (11.0%)	0.01021 ± 0.00120 (11.6%)

**Table 4:** Summary of tensile parameters of FRCM specimens subjected to water conditioning.

Series	E1 (GPa)	E2 (GPa)	σ_max_ (MPa)	ε_max_ (mm/mm)
SBRUC	221.7	176.8	1587 ± 152 (9.6%)	0.00925 ± 0.00041 (4.4%)
SBRW1000	272.5	174.7	1758 ± 114 (6.5%)	0.00987 ± 0.00072 (7.3%)
SBRW2000	217.3	173.4	1664 ± 164 (9.8%)	0.00978 ± 0.00010 (10.3%)
SBRW3000	140.3	178.8	1531 ± 95 (6.2%)	0.00964 ± 0.00078 (8.1%)
EPXUC	260.5	180.2	1958 ± 62 (3.2%)	0.01244 ± 0.00171 (13.7%)
EPXW1000	266.8	136.0	1832 ± 154 (8.4%)	0.01141 ± 0.00228 (20%)
EPXW2000	256.6	174.5	1818 ± 93 (5.1%)	0.01066 ± 0.00117 (11.0%)
EPXW3000	200.6	187.2	1854 ± 181 (9.7%)	0.01079 ± 0.00129 (11.9%)

**Table 5:** Summary of energy absorption capacity of all FRCM specimen series under different conditioning regimes.

Series	U_total_ (MPa) COV
SBRUC	7.14 ± 0.71 (9.9%)
SBRA1000	7.43 ± 1.39 (18.8%)
SBRA2000	8.36 ± 0.83 (10.0%)
SBRA3000	7.72 ± 1.95 (25.3%)
SBRW1000	8.63 ± 1.41 (16.4%)
SBRW2000	8.31 ± 1.48 (17.8%)
SBRW3000	6.77 ± 0.93 (13.8%)
EPXUC	10.84 ± 1.67 (15.4%)
EPXA1000	10.01 ± 1.37 (13.7%)
EPXA2000	11.05 ± 1.68 (15.2%)
EPXA3000	9.44 ± 1.90 (20.1%)
EPXW1000	10.67 ± 2.74 (25.6%)
EPXW2000	9.62 ± 1.64 (17.0%)
EPXW3000	9.34 ± 2.01 (21.5%)

## Data Availability

The raw data supporting the conclusions of this article will be made available by the authors on request.
